# Main Clinical Features of COVID-19 and Potential Prognostic and Therapeutic Value of the Microbiota in SARS-CoV-2 Infections

**DOI:** 10.3389/fmicb.2020.01302

**Published:** 2020-06-05

**Authors:** Yu He, Jianhui Wang, Fang Li, Yuan Shi

**Affiliations:** ^1^Department of Neonatology, Children's Hospital of Chongqing Medical University, Chongqing, China; ^2^Ministry of Education Key Laboratory of Child Development and Disorders, Chongqing, China; ^3^National Clinical Research Center for Child Health and Disorders, Chongqing, China; ^4^China International Science and Technology Cooperation Base of Child Development and Critical Disorders, Chongqing, China; ^5^Chongqing Key Laboratory of Pediatrics, Chongqing, China

**Keywords:** microbiota, COVID-19, SARS-CoV-2, ACE2, SCFAs

## Abstract

Coronavirus disease 2019 (COVID-19), caused by severe acute respiratory syndrome (SARS) coronavirus 2 (SARS-CoV-2), has become a pandemic, infecting more than 4,000,000 people worldwide. This review describes the main clinical features of COVID-19 and potential role of microbiota in COVID-19. SARS-CoV and SARS-CoV-2 have 79.5% nucleotide sequence identity and use angiotensin-converting enzyme 2 (ACE2) receptors to enter host cells. The distribution of ACE2 may determine how SARS-CoV-2 infects the respiratory and digestive tract. SARS and COVID-19 share similar clinical features, although the estimated fatality rate of COVID-19 is much lower. The communication between the microbiota and SARS-CoV-2 and the role of this association in diagnosis and treatment are unclear. Changes in the lung microbiota were identified in COVID-19 patients, and the enrichment of the lung microbiota with bacteria found in the intestinal tract is correlated with the onset of acute respiratory distress syndrome and long-term outcomes. ACE2 regulates the gut microbiota by indirectly controlling the secretion of antimicrobial peptides. Moreover, the gut microbiota enhances antiviral immunity by increasing the number and function of immune cells, decreasing immunopathology, and stimulating interferon production. In turn, respiratory viruses are known to influence microbial composition in the lung and intestine. Therefore, the analysis of changes in the microbiota during SARS-CoV-2 infection may help predict patient outcomes and allow the development of microbiota-based therapies.

## Introduction

Coronavirus disease 2019 (COVID-19), caused by severe acute respiratory syndrome (SARS) coronavirus 2 (SARS-CoV-2), initially produced a pneumonia outbreak in China and then quickly spread across the globe (The Lancet, 2020). On January 30, 2020, the World Health Organization declared the epidemic to be a public health emergency of international concern. As of May 15th, more than 4,000,000 confirmed cases and 290,000 deaths were reported worldwide (World Health Organization, 2019). The majority of studies focused on the symptoms and chest radiographic findings because SARS-COV-2 is clinically similar to SARS-CoV, which caused respiratory disease outbreaks in China in 2002 and 2003 and respiratory symptoms in 67.7–81.0% of infected patients (Zhong et al., 2003; Chen et al., 2020; Guan et al., 2020; Huang et al., 2020). In addition, previous studies reported that SARS-CoV-2 patients had digestive symptoms, including diarrhea, and test results in stools specimens or rectal swabs were positive. For this reason, the gastrointestinal (GI) tract deserves special attention because SARS-COV-2 might be transmitted via fomites (Chen et al., 2020; Guan et al., 2020). This review describes the etiology and clinical features of COVID-19 and discusses the potential role of the microbiota in disease management.

## Basic Clinical Features of COVID-19

### Etiology

Coronaviruses are genetically classified into four major genera: Alphacoronavirus, Betacoronavirus, Gammacoronavirus, and Deltacoronavirus, and infect predominantly the respiratory and intestinal tract (Li, 2016). SARS-CoV and MERS-CoV, which caused two large respiratory outbreaks in the last 20 years, belong to the genus Betacoronavirus (Drosten et al., 2003; Gomersall and Joynt, 2013). Full-length genome sequence analysis showed that SARS-CoV-2 presented a nucleotide sequence identity of 79.5% with SARS-CoV and 96% with a bat coronavirus (Zhou et al., 2020). Spike, envelope, membrane, and nucleocapsid proteins have a structural role in SARS-CoV-2 (Wu A. et al., 2020). SARS-CoV-2 is sensitive to ultraviolet radiation and heat. In addition, 75% ethanol, chlorine-containing disinfectants, and peracetic acid completely inactivate the virus (Lee, 2003).

Little is known about the genetic diversity of SARS-CoV-2. A study has shown that there may be two major strains (L and S type) based on two tightly linked SNPs. The genomic distance between these SNPs was significant, with *r*^2^ value of 0.954 and a LOD value of 50.13. The L type was more prevalent in the early phase of the outbreak in Wuhan, whereas the S type was evolutionarily older and predominated after January 2020 (Tang et al., 2020). However, the infectivity and transmissibility of different SARS-CoV-2 genotypes remain unknown.

SARS-CoV uses angiotensin-converting enzyme2 (ACE2) receptors to enter host cells (Lee and Mazmanian, 2010). Similarly, SARS-CoV-2 binds to ACE2 receptors but not to MERS-CoV receptor dipeptidyl peptidase 4 (Hoffmann et al., 2020; Wu F. et al., 2020; Zhou et al., 2020).

The origin of SARS-CoV-2 is unknown, however, bats are considered the natural reservoir because this virus is genetically similar to bat coronaviruses (Wu F. et al., 2020). Wild animals are potential intermediate hosts for SARS-CoV-2 because civet cats, which are sold in Chinese wet markets, serve as intermediate hosts for the zoonotic transmission of SARS-CoV between bats and humans, and SARS-CoV-2-infected patients in China had a history of exposure to animals sold at the Huanan Seafood Wholesale Market (Yip et al., 2009; Li et al., 2020).

### Virus Transmission

After the presumed zoonotic transmission of SARS-CoV-2 in China, evidence of human-to-human transmission was confirmed by a familial cluster of pneumonia (Hoffmann et al., 2020; Li et al., 2020). Both symptomatic and asymptomatic patients with COVID-19 can spread the virus (Rothe et al., 2020). The estimated reproductive number (R_0_) for SARS-CoV-2 varies between 2 to 3 and is higher than that for SARS-CoV (del Rio and Malani, 2020). A study found that the binding affinity of SARS-CoV-2 to ACE2 receptors is 10- to 20-fold higher than that of SARS-CoV (Wrapp et al., 2020), which may explain the higher number of COVID-19 cases relative to SARS cases.

The main routes of transmission of MERS-CoV, SARS-CoV, and SARS-CoV-2 are direct contact and respiratory droplets (Otter et al., 2016), and vertical transmission remains disputable. It is unclear whether MERS is spread via mother-to-child transmission because relevant specimens, including umbilical cord, amniotic fluid, and placenta, were not tested (Hijawi et al., 2012; Malik et al., 2016; Jeong et al., 2017). In addition, a study showed that babies born to SARS-CoV-infected mothers had no clinical and laboratory evidence of infection (Shek et al., 2003). Another study reported that nine infants born to mothers with COVID-19 had no symptoms, and the results of tests in the amniotic fluid, cord blood, neonatal throat swab, and breast milk were negative for SARS-CoV-2, confirming the absence of vertical transmission (Chan et al., 2020). Conversely, it was reported that two of six neonates born to women with COVID-19 had elevated IgG and IgM antibodies to SARS-CoV-2, although diagnostic tests for detecting the virus in the placenta, cord blood, and amniotic fluid were not performed (Zeng et al., 2020). Given that the maternal-fetal transmission of human coronaviruses is possible, large studies are necessary to confirm the vertical transmission of SARS-CoV-2 (Gagneur et al., 2008).

A cluster of SARS infection in Amoy Gardens, Hong Kong, indicated possible fomite transmission of coronaviruses because many infected patients had diarrhea (Lee, 2003) and further transmission through environmental contamination and person-to-person contact. Moreover, it has been shown that the test results of nasopharyngeal and stool samples were positive for SARS-CoV-2 before treatment and remained positive in stool or rectal samples after treatment, demonstrating that the fomite or fecal-route transmission of SARS-CoV-2 should not be ignored (Guan et al., 2020; He et al., 2020; Lingkong et al., 2020; Wang J. et al., 2020).

### Clinical Features of COVID-19

The average incubation period of COVID-19 is 3.0 days (range, 0–24.0), which is shorter than that of SARS (Supplementary Table 1; Donnelly et al., 2003; Guan et al., 2020). The most common clinical symptoms of COVID-19 and SARS are fever, fatigue, and dry cough. The average age of infected patients in different studies ranged from 45 to 56 years. Approximately 86% and more than 90% of COVID-19 and SARS patients, respectively, have abnormal chest radiographs. In addition, 6.1–32.0% of COVID-19 patients needed mechanical ventilation (Donnelly et al., 2003; Goyal et al., 2020; Guan et al., 2020). These data vary widely because different hospital protocols were used across studies (Huang et al., 2020; Young et al., 2020).

The overall case-fatality rate has not been determined because many patients are currently under treatment and follow-up. The estimated mortality in the early stage of the outbreak was 11–15% in China but does not represent the overall rate because only patients with severe symptoms were tested during this stage. In addition, the high number of asymptomatic patients limited measuring this variable accurately.

The most common complication from COVID-19 is acute respiratory distress syndrome (ARDS), which affects 3.4% of infected patients and 15.6–17.0% of severe patients (Chen et al., 2020; Guan et al., 2020). Lymphopenia is common in severe and critically ill patients and rare in patients with mild symptoms. The chest computed tomography features of COVID-19 include bilateral ground-glass opacity, consolidation, and local or bilateral patchy shadowing (Kanne, 2020; Lee, 2020).

GI symptoms are common in COVID-19 patients, and a meta-analysis showed that these symptoms occurred in 17.6% of infected patients and were more common in severe patients (Cheung et al., 2020). Similarly, approximately 25% of SARS and MERS patients had GI symptoms (Donnelly et al., 2003; Assiri et al., 2013).

### Potential Routes of SARS-CoV-2 Infection of the GI Tract

The mechanisms by how SARS-CoV-2 causes GI symptoms remain unknown. A possible route of infection is from the trachea to the esophagus since single-cell transcriptome analysis showed that ACE2 was highly expressed in lung AT2 cells, stratified epithelial cells in the upper esophagus, and enterocytes in the ileum and colon (Zhang et al., 2020). Moreover, pharyngeal swabs, esophageal biopsies, stool specimens, as well as samples from the gastric, rectal, and duodenal mucosa tested positive for SARS-CoV-2 in two patients (Guan et al., 2020). Another potential route of infection is the bloodstream because SARS-CoV-2 was detected in bleeding site in one case (Guan et al., 2020). Moreover, the expression of ACE2 in endothelial cells and macrophages, and virus detection in plasma and blood lymphocytes indicate the possibility of bloodstream infection of SARS-CoV-2 (Grant et al., 2003; Peiris et al., 2004; Wang et al., 2004; Zhao et al., 2020). However, the fecal-oral transmission of SARS-CoV-2 has not been confirmed.

## Existing Evidence About the Microbiota and SARS-COV-2

### Changes in the Microbiota in the Bronchoalveolar Lavage Fluid of COVID-19 Patients

To date, only one study analyzed changes in the composition of the lung microbiota in SARS-CoV-2-infected patients (Shen et al., 2020) and found that the microbial composition in the bronchoalveolar lavage fluid (BALF) of these patients was different from that of healthy controls and was dominated by either pathogenic bacterial strains or commensal bacteria commonly found in the oral and upper respiratory tract. In addition, this microbial composition was similar to that of patients with community-acquired pneumonia. However, the microbial signature associated with SARS-CoV-2 was similar to that of other respiratory viruses such as influenza and respiratory syncytial virus (RSV). Notwithstanding, this conclusion was limited by the small sample size (eight patients) (Shen et al., 2020). Few studies have examined the association between lower respiratory tract (LRT) microbiota and viral infections. There was an increase in the abundance of *Streptococcus* and *Staphylococcus* in the BALF of H1N1-infected mice and in the abundance of *H. influenzae* in rhinovirus-infected patients with chronic obstructive pulmonary disease (Molyneaux et al., 2013; Gu et al., 2019). Changes in the microbiota in the LRT during viral infection were variable and might be a result of the reduced ability to clear pathogens in the upper respiratory tract.

### Relationship Between Coronavirus, ACE2, and the Gut Microbiota

**Figure 1:**
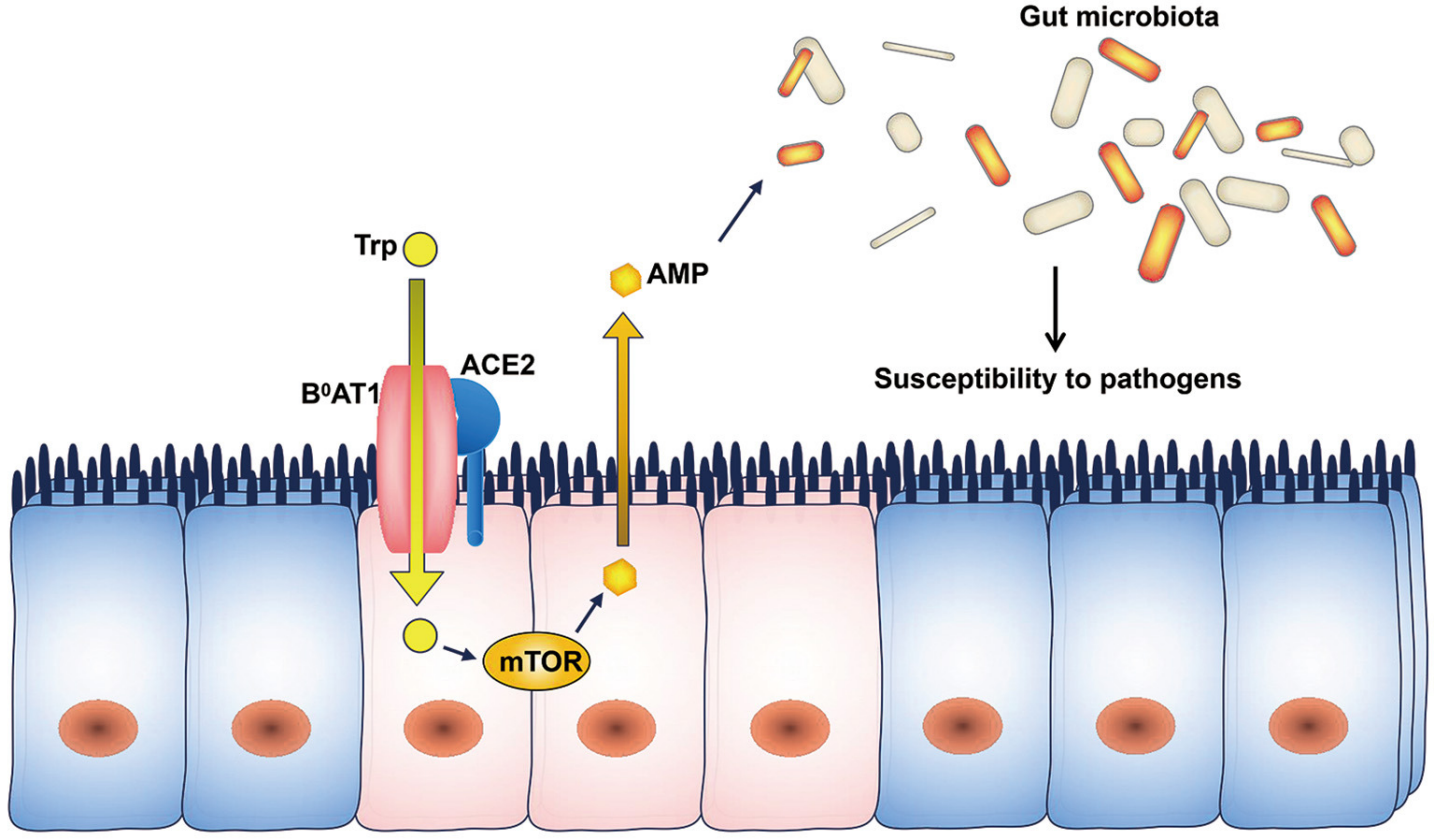
ACE2 and the microbiota. The intestinal uptake of tryptophan is mediated by B^0^AT1, and ACE2 is indispensable for the expression of B^0^AT1. Tryptophan stimulates the secretion of antimicrobial peptides through the mTOR pathway. Changes in the levels of antimicrobial peptides can influence the composition of the gut microbiota. AMP, antimicrobial peptides; Trp, tryptophan.

ACE2 expression is downregulated in SARS patients during infection (Kuba et al., 2005). ACE2 regulates the expression of the amino acid transporter B^0^AT1, which controls the intestinal uptake of tryptophan (Hashimoto et al., 2012). Tryptophan regulates the mRNA expression of antimicrobial peptides through the mTOR pathway (Zhao et al., 2018), and antimicrobial peptides may influence the composition of the gut microbiota (Lievin-Le Moal and Servin, 2006). As a result, ACE2 downregulation decreases the intestinal absorption of tryptophan and reduces the secretion of antimicrobial peptides, leading to increased pathogen survival and gut dysbiosis (Figure 1). Therefore, the ACE2-dependent regulation of the microbiota may explain the occurrence of diarrhea in SARS-CoV and SARS-CoV-2 infections.

## Potential Role of the Microbiota in the Prognosis and Treatment of COVID-19

### The Presence of Gut Microbes in the Lung May Predict ARDS

ARDS is a common and severe complication of COVID-19, and evidence shows that the lung microbiota of patients with ARDS is different from that of patients without ARDS; therefore, changes in the microbial composition in the lung of COVID-19 patients may predict ARDS (Meyer and Calfee, 2017; Panzer et al., 2018; Kyo et al., 2019; Dickson et al., 2020). Dickson et al. used high-throughput sequencing to identify the microbiota in the BALF of 68 patients with ARDS. The results showed that gut-associated *Bacteroides* species were present in 41% of patients vs. 3.8% of healthy controls, and the enrichment of the lung microbiota with gut bacteria was correlated with elevated inflammatory markers in plasma (Dickson et al., 2016). Another study demonstrated that the abundance of gut-associated *Enterobacteriaceae* spp. was increased in critically ill patients with ARDS compared with patients without ARDS. In addition, the increased number of gut-associated *Lachnospiraceae* and *Enterobacteriaceae* predicted fewer ventilator-free days, and an increase in *Lachnospiraceae* was a strong predictor of reduced survival in ARDS patients (Dickson et al., 2020). These results suggest that the microbiota can be used as a marker to predict ARDS and the outcomes of COVID-19.

### Microbiota and Virus Infection

Both the innate and adaptive immune systems are involved in SARS-CoV-2 infection. Lymphopenia with drastically reduced numbers of B cells, CD4^+^ and CD8^+^ T cells, and monocytes, and the upregulation of programmed cell death-1, a biomarker of T-cell exhaustion, occur in severe COVID-19 patients (Cao, 2020; Diao et al., 2020). In addition, the microbiota modulates the immune system (Round et al., 2011; Cebula et al., 2013) by affecting the development of immune cells, such as regulatory T cells and innate lymphoid cells, which help maintain gut and lung homeostasis (Furusawa et al., 2013; Smith et al., 2013; Hepworth et al., 2015). Although the data on the interaction between normal microbiota and viruses are limited, accumulating evidences with different interventions such as antibiotic exposure and microbiota transfer showed that the microbiota enhanced antiviral immunity (Table 1). These findings may allow developing effective therapies for SARS-CoV-2 infection.

**Table 1:** Summary of antiviral functions of the gut microbiota.

**Bacterial species**	**Intervention**	**Microbial factors**	**Mechanisms**	**Response**	**References**
Commensal microbiota	Antibiotic exposure	Undefined	• Inflammasome-mediated migration of DCs and specific CD8^+^T cell priming • Protection against viral infections and enhancement of IFN signaling in macrophages	Anti-influenza	Ichinohe et al., 2011; Abt et al., 2012
Increased abundance of *Bacteroides* species	HFD SCFA treatment	SCFAs (butyrate)	• Enhancement of CD8^+^T cell metabolism • Increased generation of macrophages with reduced ability to produce CXCL1 in airways • Reduced neutrophil recruitment, resulting in the attenuation of lung immunopathology	Anti-influenza	Trompette et al., 2018
*Clostridium orbiscindens*	Antibiotic exposure	DAT	Enhanced type I IFN signaling in macrophages	Anti-influenza	Steed et al., 2017
Commensal microbiota	Antibiotic exposure Microbiota transfer	Undefined	Production of virus-specific CD8^+^T cell responses via DCs	Anti-West Nile virus	Thackray et al., 2018
*Lachnospiraceae* spp. (phylum Firmicutes, class Clostridia)	HFD SCFA treatment Antibiotic exposure	SCFAs (acetate)	GPR43-mediated and IFNAR dependent IFN-β responses in lung epithelial cells	Anti-RSV	Antunes et al., 2019

*DAT, desaminotyrosine; DCs, dendritic cells; HFD, high-fiber diet; IFN, interferon; RSV, respiratory syncytial virus; SCFAs, short-chain fatty acids*.

Mice treated with antibiotics had impaired anti-influenza immunity. The normal gut microbiota can active the inflammasome and induce the migration of dendritic cells to initiate T-cell responses to the influenza virus and activate antiviral responses in macrophages (Ichinohe et al., 2011; Abt et al., 2012). It has been reported that antibiotic exposure impaired West Nile virus-specific CD8^+^ T-cell responses and increased infection and immunopathology (Thackray et al., 2018). Although these results demonstrate the antiviral role of the microbiota, the direct association between the microbiota and virus-specific immune cells is unknown. Microbial metabolites regulate the host immune system (Hooper et al., 2012). Short-chain fatty acids (SCFAs) and desaminotyrosine produced by *Bacteroidetes and/or Clostridium* can enhance influenza-specific CD8^+^ T-cell function and type I interferon (IFN) signaling in macrophages, increasing protection against influenza infection (Atarashi et al., 2013; Tanoue et al., 2016; Steed et al., 2017; Trompette et al., 2018). Influenza-infected mice fed a high-fiber diet exhibited changes in the microbiota, with increased production of SCFAs and increased differentiation of Ly6c^−^ patrolling monocytes in the bone marrow, limiting the synthesis of the chemokine CXCL1 in the airways, leading to the suppression of neutrophil recruitment to the airways and attenuation of lung immunopathology (Trompette et al., 2018). Similarly, a high-fiber diet increased the relative abundance of SCFA-producing *Lachnospiraceae* spp. The SCFA acetate protected mice against RSV infection through IFN-β production in lung epithelial cells via G-protein-coupled receptors (Antunes et al., 2019). Given that lymphopenia is common in COVID-19 patients and probiotics can improve protection against influenza infection, the microbiota can potentially serve as a target for antiviral therapy (Maeda et al., 2009; Wang D. et al., 2020).

Respiratory viruses can also change the composition of the gut microbiota. It has been shown that the abundance of *Proteobacteria* and *Bacteroidetes* is increased, whereas the abundance of *Firmicutes* is decreased during influenza and RSV infections. The influence of these viruses on the gut microbiota may be mediated by systemic signals, including types I and II IFN, physiologic changes, and increased susceptibility to colitis (Deriu et al., 2016; Bartley et al., 2017; Groves et al., 2018).

These data suggest that the microbiota improves antiviral immunity and may play a role in SARS-CoV-2 infection. A clinical trial on microbiota transplantation in COVID-19 patients is ongoing (Zhang, 2020); notwithstanding, additional studies are necessary to elucidate this role.

## Discussion

This review described the epidemiological features of SARS-CoV-2 and COVID-19 and investigated the potential role of the microbiota in SARS-CoV-2 infection. The microbiota signature in the lung may predict ARDS and long-term outcomes in COVID-19 patients. Diarrhea during SARS-CoV-2 infections should not be ignored, and the dysregulation of ACE2 expression may contribute to gut dysbiosis. In addition, understanding how changes in microbial communities promote viral infections may allow developing effective therapies for this novel coronavirus.

As COVID-19 has rapidly spread throughout the world, health workers, epidemiologists, and scientists should work together to address three issues: (1) determine the virulence and fatality rate of different SARS-CoV-2 genotypes in different geographic areas and the relationship between these genotypes and epidemiology; (2) investigate the potential mechanism by which SARS-CoV-2 attacks the immune system considering that ACE2 expression is low in T and B cells, and analyze how lymphopenia predicts disease severity; (3) understand how the microbiota can help assess clinical status and serve as a target for anti-SARS-CoV-2 therapies.

## Author Contributions

YH, FL, and YS conceptualized the manuscript, wrote the first draft, and edited subsequent versions. YH, JW, and YS contributed ideas on the texts. All authors read and approved the final manuscript.

## Conflict of Interest

The authors declare that the research was conducted in the absence of any commercial or financial relationships that could be construed as a potential conflict of interest.
