# Supplementary material for: Main Clinical Features of COVID-19 and Potential Prognostic and Therapeutic Value of the Microbiota in SARS-CoV-2 Infections
Source: Front Microbiol. 2020 Jun 5;11:1302. doi: 10.3389/fmicb.2020.01302 (PMC7291771; doi:10.3389/fmicb.2020.01302)
Supplement: Supplementary file 1 [file Table_1.docx]

| Supplementary Table 1. Clinical features of COVID-19 and SARS. | | | | | | | |
| --- | --- | --- | --- | --- | --- | --- | --- |
| Items | COVID-19  (Huang et al., 2020) | COVID-19  (Chen et al., 2020) | COVID-19  (Wang et al., 2020) | COVID-19  (Guan et al., 2020) | COVID-19  ([Young et al., 2020](#_ENREF_2)) | COVID-19  ([Goyal et al., 2020](#_ENREF_1)) | SARS  (Donnelly et al., 2003) |
| Journal | Lancet | Lancet | JAMA | NEJM | JAMA | NEJM | Lancet |
| Number of cases | 41 | 99 | 138 | 1099 | 18 | 393 | 1425 |
| Publication date | 2020/1/24 | 2020/1/29 | 2020/2/7 | 2020/2/6 | 2020/3/3 | 2020/4/17 | 2003/3/24 |
| Population origin | Wuhan, China | Wuhan, China | Wuhan, China | China | Singapore | New York, USA | Hong Kong, China |
| Incubation period (median) | NA | NA | NA | 3.0 days | NA | NA | 6.4 days |
| Fever(%) | 98 | 83 | 98.6 | 87.9 | 72.2 | 77.1 | 94 |
| Cough(%) | 76 | 82 | 82 | 67.7 | 83.3 | 79.4 | 50.4 |
| Shortness of breath(%) | 55 | 31 | 31.2 | 18.6 | 11.1 | 56.5 | 30.6 |
| Sputum production(%) | 28 | NA | 26.8 | 33.4 | NA | NA | 27.8 |
| Diarrhea(%) | 3 | 2 | 10.1 | 3.7 | 16.6 | 23.7 | 27 |
| Death(%) | 15 | 11 | 4.3 | 1.4 | 0 | 10.2 | <60 years old: 13.2%  >60 years old: 43.3% |
| COVID-19, coronavirus disease 2019; SARS, severe acute respiratory syndrome | | | | | | | |

References：

# Chen, N., Zhou, M., Dong, X., Qu, J., Gong, F., Han, Y., Qiu, Y., Wang, J., Liu, Y., Wei, Y., Xia, J.A., Yu, T., Zhang, X., and Zhang, L. (2020). Epidemiological and clinical characteristics of 99 cases of 2019 novel coronavirus pneumonia in Wuhan, China: a descriptive study. *The Lancet.* 395, 507-513.

# Donnelly, C.A., Ghani, A.C., Leung, G.M., Hedley, A.J., Fraser, C., Riley, S., Abu-Raddad, L.J., Ho, L.-M., Thach, T.-Q., Chau, P., Chan, K.-P., Lam, T.-H., Tse, L.-Y., Tsang, T., Liu, S.-H., Kong, J.H.B., Lau, E.M.C., Ferguson, N.M., and Anderson, R.M. (2003). Epidemiological determinants of spread of causal agent of severe acute respiratory syndrome in Hong Kong. *The Lancet.* 361, 1761-1766.

Goyal, P., Choi, J.J., Pinheiro, L.C., Schenck, E.J., Chen, R., Jabri, A., Satlin, M.J., Campion, T.R., Nahid, M., Ringel, J.B., Hoffman, K.L., Alshak, M.N., Li, H.A., Wehmeyer, G.T., Rajan, M., Reshetnyak, E., Hupert, N., Horn, E.M., Martinez, F.J., Gulick, R.M., and Safford, M.M. (2020). Clinical Characteristics of Covid-19 in New York City. *New England Journal of Medicine*. DOI: 10.1056/NEJMc2010419

# Guan, W.-J., Ni, Z.-Y., Hu, Y., Liang, W.-H., Ou, C.-Q., He, J.-X., Liu, L., Shan, H., Lei, C.-L., Hui, D.S.C., Du, B., Li, L.-J., Zeng, G., Yuen, K.-Y., Chen, R.-C., Tang, C.-L., Wang, T., Chen, P.-Y., Xiang, J., Li, S.-Y., Wang, J.-L., Liang, Z.-J., Peng, Y.-X., Wei, L., Liu, Y., Hu, Y.-H., Peng, P., Wang, J.-M., Liu, J.-Y., Chen, Z., Li, G., Zheng, Z.-J., Qiu, S.-Q., Luo, J., Ye, C.-J., Zhu, S.-Y., and Zhong, N.-S. (2020). Clinical characteristics of coronavirus disease 2019 in China. *New England Journal of Medicine*.DOI: 10.1056/NEJMoa2002032

# Huang, C., Wang, Y., Li, X., Ren, L., Zhao, J., Hu, Y., Zhang, L., Fan, G., Xu, J., Gu, X., Cheng, Z., Yu, T., Xia, J., Wei, Y., Wu, W., Xie, X., Yin, W., Li, H., Liu, M., Xiao, Y., Gao, H., Guo, L., Xie, J., Wang, G., Jiang, R., Gao, Z., Jin, Q., Wang, J., and Cao, B. (2020). Clinical features of patients infected with 2019 novel coronavirus in Wuhan, China. *The Lancet.* 395, 497-506.

Wang, L.-F., Tan, B.H., Lin, R.T.P., Lee, V.J.M., Leo, Y.-S., Lye, D.C., and Team, F.T.S.N.C.O.R. (2020). Epidemiologic Features and Clinical Course of Patients Infected With SARS-CoV-2 in Singapore. *JAMA* 323, 1488-1494. DOI: 10.1001/jama.2020.3204

Young, B.E., Ong, S.W.X., Kalimuddin, S., Low, J.G., Tan, S.Y., Loh, J., Ng, O.-T., Marimuthu, K., Ang, L.W., Mak, T.M., Lau, S.K., Anderson, D.E., Chan, K.S., Tan, T.Y., Ng, T.Y., Cui, L., Said, Z., Kurupatham, L., Chen, M.I.-C., Chan, M., Vasoo, S., Wang, L.-F., Tan, B.H., Lin, R.T.P., Lee, V.J.M., Leo, Y.-S., Lye, D.C., and Team, F.T.S.N.C.O.R. (2020). Epidemiologic Features and Clinical Course of Patients Infected With SARS-CoV-2 in Singapore. *JAMA* 323, 1488-1494. DOI: 10.1001/jama.2020.3204
